# Labeled vs. Label-Free Raman Imaging of Lipids in Endothelial Cells of Various Origins

**DOI:** 10.3390/molecules25235752

**Published:** 2020-12-06

**Authors:** Basseem Radwan, Adriana Adamczyk, Szymon Tott, Krzysztof Czamara, Katarzyna Kaminska, Ewelina Matuszyk, Malgorzata Baranska

**Affiliations:** 1 Jagiellonian Centre for Experimental Therapeutics (JCET), Jagiellonian University, 14 Bobrzynskiego Str., 30-348 Krakow, Poland; basseem.radwan@uj.edu.pl (B.R.); adriana.adamczyk@doctoral.uj.edu.pl (A.A.); szymon.tott@doctoral.uj.edu.pl (S.T.); krzysztof.czamara@uj.edu.pl (K.C.); 2 Faculty of Chemistry, Jagiellonian University, 2 Gronostajowa Str., 30-387 Krakow, Poland; katarzyna1.kaminska@uj.edu.pl

**Keywords:** endothelial cells, astaxanthin, lipids, Raman spectroscopy

## Abstract

Endothelial cells (EC) constitute a single layer of the lining of blood vessels and play an important role in maintaining cardiovascular homeostasis. Endothelial dysfunction has been recognized as a primary or secondary cause of many diseases and it manifests itself, among others, by increased lipid content or a change in the lipid composition in the EC. Therefore, the analysis of cellular lipids is crucial to understand the mechanisms of disease development. Tumor necrosis factor alpha (TNF-α)-induced inflammation of EC alters the lipid content of cells, which can be detected by Raman spectroscopy. By default, lipid detection is carried out in a label-free manner, and these compounds are recognized based on their spectral profile characteristics. We consider (3S,3′S)-astaxanthin (AXT), a natural dye with a characteristic resonance spectrum, as a new Raman probe for the detection of lipids in the EC of various vascular beds, i.e., the aorta, brain and heart. AXT colocalizes with lipids in cells, enabling imaging of lipid-rich cellular components in a time-dependent manner using laser power 10 times lower than that commonly used to measure biological samples. The results show that AXT can be used to study lipids distribution in EC at various locations, suggesting its use as a universal probe for studying cellular lipids using Raman spectroscopy. The use of labeled Raman imaging of lipids in the EC of various organs could contribute to their easier identification and to a better understanding of the development and progression of various vascular diseases, and it could also potentially improve their diagnosis and treatment.

## 1. Introduction

Endothelial cells (EC) constitute a single layer that lines blood and lymph vessels and directs the selective movement of substances between the blood and surrounding cells. The endothelium plays an important role in various physiological processes that are responsible for the body’s homeostasis [1]. EC mediate vasodilatation, prevent platelet adhesion and activation, block thrombin formation and mitigate fibrin deposition. However, a variety of factors can cause endothelial dysfunction (ED) and lead to the development of cardiovascular diseases, such as chronic heart failure, peripheral arterial disease, atherosclerosis, and many others. The common factor of these disorders is the inflammation of the blood vessels, which appears to have a profound effect on the endothelium [1].

Stimulation with tumor necrosis factor alpha (TNF-α), a pleiotropic cytokine considered an important contributor to ED, is one of the most widely used in vitro model of inflammation. TNF-α is produced mostly by inflammatory cells which play an important role in cell survival, proliferation, differentiation, and death [2]. As a result of ED, several modifications visible in ultrastructural studies are observed. One of the most significant cellular changes associated with ED is the disturbance of lipid amount and distribution [3].

The abnormal lipid accumulation leads to lipotoxicity, which might be a cause of various pathogenesis. It is well known that cardiovascular diseases, i.e., atherosclerosis or coronary heart disease, are related to lipotoxicity. Moreover, lipid accumulation can cause oxidative stress and cell death [4]. It was also reported that failure in gluconeogenesis is caused by excessively accumulated lipids [5]. Lipid-rich cellular organelles called lipids droplets (LDs) have long been known to dynamically respond to inflammatory stimuli. LDs are involved in the mediation and coordination of lipid metabolism with cellular requirements for energy production, membrane homeostasis and cell growth [6]. Literature reports indicate that the inflammatory process promotes LDs formation in EC [7]. Therefore, sensitive and selective methods to measure lipid content and distribution in cells are of great importance. Raman spectroscopy has previously been used to study the LDs composition of the EC under normal conditions and during disease development [8,9]. Abnormalities in the composition, quantity or size of LDs in EC have been recognized as one of the markers of ED, in addition to other lipid-related markers, such as the lipid-to-protein ratio [10]. While Raman spectra of cells are complex, Raman spectroscopy is still widely used in cell studies due to its ability to provide insight into chemical structures of important cellular molecules. For instance, visualizing cell nuclei is possible based on the Raman peak at ca. 785 cm^−1^ assigned to the ring breathing modes of DNA and RNA bases, and tracking protein distribution in the cells could be performed by integration of the band at ca. 1005 cm^−1^ assigned to phenylalanine ring breathing modes. Moreover, lipids can be detected by Raman spectroscopy based on their characteristic Raman bands that are observed in the following spectral regions: 3000–2800, 1500–1400, 1300–1250 and 1200–1050 cm^−1^ assigned to the C–H stretching, scissoring of the CH_2_ group, twisting of the CH_3_ group and C–C stretching vibrations, respectively [11]. Although, other biomolecules in EC exhibit some peaks in the mentioned spectral regions, Raman detection of lipids is conducted relatively easily due to their long hydrocarbon chains resulting in their large scattering cross-section [11]. Therefore, it makes Raman spectroscopy a reliable tool to study lipids in EC and their association with ED without the need of using dyes. This label-free approach is advantageous over other methods that rely on dyes, which can be cytotoxic and eventually may alter the outcome of the experiment. Nonetheless, recent studies have shown the potential of using molecular reporters that are specific to certain subcellular structures to improve the quality of information obtained from Raman measurements [12,13,14]. Generally, when we compare both approaches, labeled versus label-free Raman imaging, an advantage of the latter method is the fact that no substances are introduced into the cells. Moreover, cells measured in this way do not require additional time for stimulation or incubation with a reporter molecule. On the other hand, some fragile cells or tissues need to be tested under special conditions. Too-high laser power may lead to local overheating and damage of the sample. Moreover, the diffraction limit for some subcellular structures is too low to be represented if the Raman signal is not strong enough. Generally, Raman reporters that show intense bands or those well separated from one of the other cellular components can be used to selectively image subcellular structures.

Recently, we presented a new Raman reporter for cellular studies on lipids detection and distribution, i.e., astaxanthin (3,3′-dihydroxy-β,β′-carotene-4,4′-dione, AXT) [15]. It is a lipid-soluble keto-carotenoid from the class of terpenes, which is naturally produced by algal species such as *Haematococcus pluvialis, Chlorella zofingiensis, Chlorococcum* and *Phaffia rhodozyma* [16,17]. AXT contains a chromophore group, a long polyene chain of eleven conjugated C=C bonds, including two in the terminal rings, absorbing light in the visible range that coincides with the excitation of 532 nm lasers commonly used in Raman imaging of biological samples. Depending on the laser, resonance or pre-resonance Raman spectrum of AXT is dominated by bands at ca. 1520, 1159 and 1009 cm^−1^ due to the C=C stretching, C–C stretching and CH_3_ group wagging vibrations, respectively [18]. We previously proved for the human dermal microvascular EC (HMEC-1) that AXT is accumulating in specific lipidic structures, i.e., mainly in LDs and endoplasmic reticulum (ER). This finding revealed that AXT can be used as a selective biosensor for lipidic structures for Raman imaging studies [15].

Considering the above, our present work was aimed to answer the following question: is AXT really a universal Raman reporter for studying lipids in EC? The concentration and distribution of lipids in healthy and inflamed EC may be different. Thus, we performed experiments with AXT on cells under normal conditions and on the cells treated with TNF-α to induce inflammation. Additionally, we aimed to compare Raman imaging of lipids in labeled and label-free approaches, considering that the endothelium from various vascular beds could be structurally, functionally and chemically heterogeneous. Thus, in pathological conditions, the severity of the defects and the degree of changes in organelles arising from cellular mechanisms may also be heterogeneous. In this work, we compare the lipid distribution in different human aortic endothelial cells (HAoEC), human brain microvascular endothelial cells (HBEC-5i) and human coronary artery endothelial cells (HCAEC) derived from the aorta, brain, and heart, respectively.

## 2. Results and Discussion

### 2.1. Visualization of Cellular Lipids In Vitro in Endothelial Cells

**Figure 1:**
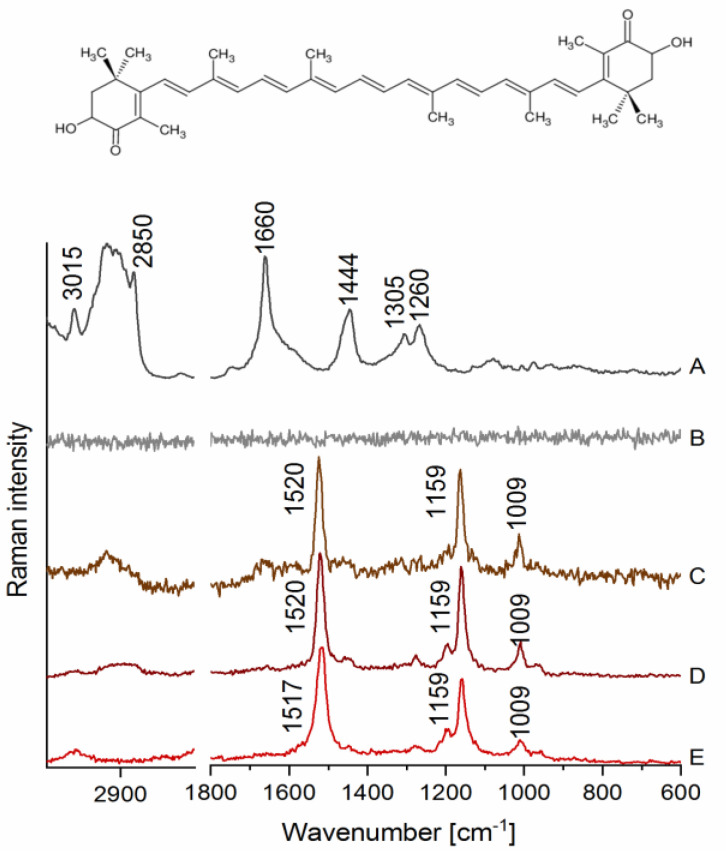
Raman spectra of lipids droplets (LDs) in human aortic endothelial cells (HAoEC) and (3S,3′S)-astaxanthin (AXT) in various environments. (**A**) A spectrum collected from an LD measured at 30 mW laser power, (**B**) a spectrum from an LD measured at 3 mW laser power, (**C**) a spectrum from an LD of the cell treated with AXT (6 h) measured at 3 mW laser power, (**D**) a spectrum of AXT dissolved in castor oil, and (**E**) a spectrum of AXT in the solid state with the AXT chemical structure on the top.

Raman spectra of LDs in HAoEC measured at ca. 3 mW (low) and 30 mW (high) laser power, as well as a spectrum of AXT in various environments, are presented in Figure 1. As shown in Figure 1A, characteristic bands for lipids were observed ca. 3015 cm^−1^ (stretching of =C–H), 2850 cm^−1^ (C–H stretching), 1660 cm^−1^ (C=C stretching), 1444 cm^−1^ (scissoring of the -CH_2_ group), 1305 cm^−1^ (twisting of the CH_2_/CH_3_ groups) and 1260 cm^−1^ (C–H bending) when EC were measured using ca. 30 mW laser power. However, when cells were measured using relatively low laser power (3 mW), it was not possible to obtain any information about lipids, as the spectrum was dominated by noise (Figure 1B), proving that detection of lipids requires higher laser power, which may not be suitable for more fragile biological samples, i.e., live cells. On the other hand, very distinct bands were observed in cells treated with AXT, e.g., at ca. 1520 cm^−1^ (C=C stretching), 1159 cm^−1^ (C–C stretching) and 1009 cm^−1^ (CH_3_ group wagging), all of them assigned to AXT (Figure 1C). Furthermore, due to the high affinity of AXT towards lipids and the colocalization of AXT with intracellular lipidic structures, it was possible to image lipids in EC samples using laser power lower 10 times.

AXT is known to change its conformation in different environments also due to its tendency to form aggregates, which results in a shift in the most intense band arising from C=C stretching of AXT in the Raman spectrum [19]. We measured Raman spectrum of AXT in LDs of EC and compared it with a reference one of AXT in the solid state and when dissolved in castor oil, a lipidic solvent for which AXT has high affinity [20]. The bands assigned to AXT in LDs (Figure 1C) clearly align with the bands observed in the spectrum of AXT measured in castor oil (Figure 1D), where both showed the most intense signal at ca. 1520 cm^−1^. In contrast, the spectrum of AXT in the solid state (Figure 1E) exhibits the band at ca. 1517 cm^−1^, which may demonstrate a different form of AXT than that observed for AXT in LDs of EC.

Another advantage of AXT as a probe for visualization of lipids is that it does not contribute to the samples’ inflammation but rather has a minimal anti-inflammatory effect on the EC in the used concentration and incubation times, as shown in a previous study by checking the level of intracellular adhesion molecule-1 (ICAM-1) expression in HMEC-1 cells, with and without AXT treatment [15]. These features allow the use of AXT to detect lipids in the EC, both under normal conditions and when it is inflamed, as AXT will not increase cells’ inflammation state.

**Figure 2:**
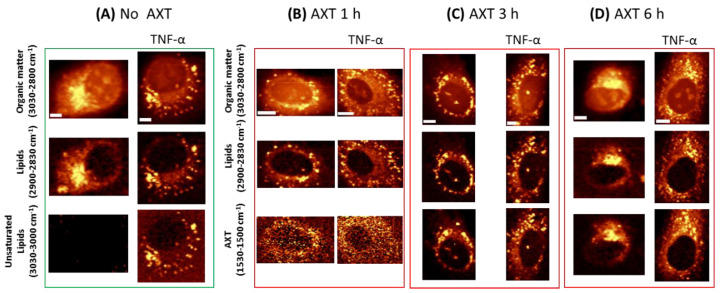
Representative Raman images of HAoEC cells: without and with AXT treatment and treated with tumor necrosis factor alpha (TNF-α) (**A**),cells treated with tumor necrosis factor alpha (TNF-α) (right part of each panel) followed by AXT stimulation for 1 h (**B**), 3 h (**C**) and 6 h (**D**), obtained by the integration of Raman bands over the selected spectral regions: 3030–2830 cm^−1^ (organic matter), 2900–2850 cm^−1^ (lipids), 3030–3000 cm^−1^ (unsaturated lipids) and 1530–1500 cm^−1^ (AXT). Scale bars equal 5 µm.

Raman images of HAoEC, under their normal state and upon inflammation (due to treatment with pro-inflammatory cytokine TNF-α) followed by incubation with AXT, are shown in Figure 2. Images displaying the distribution of lipids were obtained by the integration of respective Raman bands at 2900–2830 cm^−1^ associated with C–H stretching of lipids (Figure 2), and at 1530–1520 cm^−1^ representing the most intense band of AXT as a marker of the molecule’s distribution in the cells during different incubation times. The acquired images show the time dependency of AXT’s accumulation in EC. AXT is visible in the cytoplasm after short incubation time (1 h); however, as the incubation time increases (3 h, 6 h) AXT accumulation in lipid-rich cellular structures (i.e., LDs) is seen more clearly. Moreover, after 6 h of incubation, AXT is spread in the whole cytoplasm and accumulated around the nucleus, with the highest intensity at cellular lipidic structures, i.e., LDs and ER.

As previously shown, EC when stimulated with TNF-α produce more LDs composed of more unsaturated lipids [8]. As shown in Figure 2, Raman imaging clearly demonstrates LDs formed after TNF-α treatment, and unsaturated lipids (3030–3000 cm^−1^) are clearly seen as a fraction of all lipids in the cells.

Due to the lipophilic properties of AXT, it shows higher accumulation in cells that possess more lipids. Consequently, Raman imaging shows increased intensity of AXT bands and relatively higher accumulation of AXT in cells pre-treated with TNF-α compared to those that were not stimulated with TNF-α.

**Figure 3:**
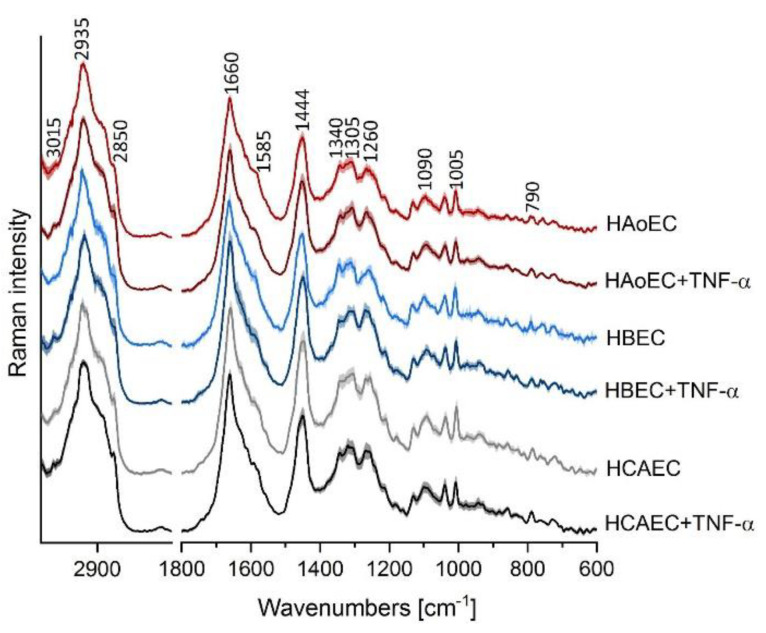
Raman spectral profiles of endothelial cells of various origin. Averaged Raman spectra from all measurements with a standard deviation at each data point of HAoEC (red), human brain microvascular endothelial cells (HBEC-5i, blue) and human coronary artery endothelial cells (HCAEC, gray) as control cells and after TNF-α treatment (darker colored spectra).

Cluster analysis (CA, *k*-means with Manhattan distance) was used to extract mean spectra of cells (*n* = 5 in each group) from HAoEC, HBEC-5i and HCAEC as the control group, and after treatment with TNF-α (24 h) as models of inflamed cells (TNF-α group). As shown in Figure 3, all normal EC from different organs show similar Raman profiles, whereas the TNF-α group of cells exhibits a trend of an elevated level of unsaturated lipids manifested by a higher intensity band at ca. 3015 cm^−1^*,* especially in the case of the HBEC-5i cell line. This finding is in agreement with previous research on the response of EC to stimulation by TNF-α studied by using Raman spectroscopy [8]. Moreover, EC lines appear to react similarly to TNF-α treatment regardless of the organ from which they are collected.

### 2.2. Endothelial Cells of Different Vascular Beds Visualized after Treatment of Astaxanthin

EC of different origin, human aortic endothelial cells (HAoEC), human brain microvascular endothelial cells (HBEC-5i) and human coronary artery endothelial cells (HCAEC), were subjected to the same treatment and measured under the same conditions after stimulation of AXT cells in their normal and inflamed state.

In general, Raman images of investigated cells suggest that all of them contain relatively high amount of lipids distributed within the whole cytoplasm, ER, and LDs. Distribution of AXT visualized under low laser power generally colocalizes with images of lipids that were measured with ten times higher laser power. However, after 1 h incubation of cells with AXT, there is no clear colocalization between AXT and lipids for HBEC-5i, while much better correlation can be noticed for HAoEC. After 3 and 6 h incubation with AXT, the images of AXT distribution and lipids are almost identical. Still, the first ones are collected under low laser power. It is worth mentioning that longer incubation time of cells with AXT exhibits improved visibility of LDs in contrast to lipids occurring in the cytoplasm, ER and other lipidic structures.

**Figure 4:**
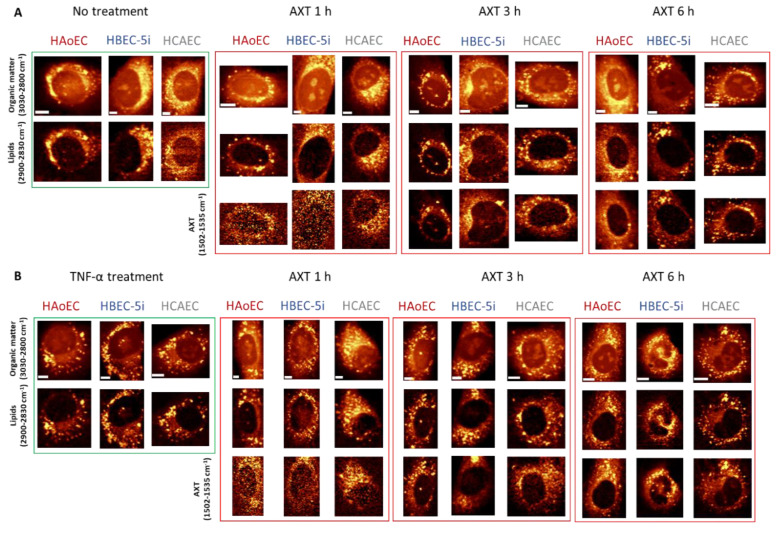
Representative Raman images of non-treated (**A**) and TNF-α-stimulated (**B**) endothelial cells incubated with AXT for 1, 3 and 6 h. Raman images of endothelial cell lines HAoEC, HBEC-5i and HCAEC were obtained by the integration of Raman bands over the selected spectral regions: 3030–2830 cm^−1^ (organic matter), 2900–2850 cm^−1^ (lipids) and 1530–1500 cm^−1^ (AXT). Scale bars equal 5 µm.

It is clear that all three EC lines responded similarly to TNF-α stimulation, i.e., cells stimulated with TNF-α (Figure 4B) show increased number of lipid entities (i.e., LDs) spread over the cytoplasm compared to control cells (Figure 4A), consequently, showing clearer localization of LDs. The colocalization of AXT and intracellular lipids (Figure 4) is independent of the EC origin, i.e., distribution of cellular lipidic structures (i.e., LDs and ER) could be interpreted based on the distribution of AXT and images collected with 10 times lower laser power applied to EC of different organs (aorta, brain, heart).

## 3. Materials and Methods 

### 3.1. Cell Culture

In this study, three cell line were chosen as models of endothelial cells originating from the aorta, brain and heart. Primary human aortic endothelial cells (HAoEC) were cultured in supplemented endothelial growth medium EGM-2MV (Lonza, Basel, Switzerland). Human brain microvascular endothelial cells (HBEC-5i) were cultured in DMEM (Dulbecco’s Modified Eagle Medium):F12 (ATCC, Manassas, VA, USA) medium supplemented with 10% fetal bovine serum (FBS, Gibco-BRL Life Technologies, Waltham, MA, USA) and 1% of antibiotics (streptomycin, penicillin and fungison, Gibco Life Technologies) and endothelial cells growth supplement (ECGS, ATTC) at a concentration of 40 µg/mL. For culturing of human coronary artery endothelial cells (HCAEC), the complete EGM-2MV medium was used supplemented with 10 mM L-glutamine (Gibco Life Technologies), 1 μg/mL hydrocortisone (Sigma Aldrich, St. Louis, MO, USA), 10 mg/mL epidermal growth factor (EGF, Sigma Aldrich), 10% fetal bovine serum (FBS, Gibco Life Technologies) and 1% of antibiotics (streptomycin, penicillin and fungison, Gibco Life Technologies). The cell cultures were incubated in a 37 °C, 5% CO_2_/95% air humidified cell culture incubator. For the experiments, HAoEC, HBEC-5i and HCAEC cells were seeded directly onto CaF_2_ windows and in the amount of approximately 120,000 per slide. After sufficient time for cells to proliferate and spread had passed, enabling them to reach the optimal confluency, half of the samples were stimulated for the next 24 h with 10 ng/mL human tumor necrosis factor alpha (TNF-α, Sigma Aldrich). At the same time, the remaining cell samples were held in the fresh culture medium. In the next step, all cells, except for a control group, were treated, dissolved in DMSO and suspended in 10 µM of (3S,3′S)-astaxanthin (AXT, Sigma Aldrich) medium for 1, 3 and 6 h. For each time of incubation with AXT, separate samples were prepared. Afterwards, fixation with the 2.5% of glutaraldehyde for 4 min was performed. Fixed cells were maintained in phosphate buffer saline (PBS, Gibco Life Technologies) and stored at a constant temperature of 4 °C. 

### 3.2. Raman Imaging

Raman imaging of all samples was performed using a WITec alpha 300 Confocal Raman Imaging system (WITec GmbH, Ulm, Germany) equipped with a Ultra-High-Throughput Screening (UHTS) 300 spectrograph, a Charge-coupled device (CCD) detector (Andor, DU401A-BV-352) and a 60× water immersion objective (Nikon Fluor, NA = 1.0). The air-cooled solid-state laser with an excitation wavelength of 532 nm was used to excite the samples with a laser power at the cell samples of 3 mW (low laser power) and 30 mW (high laser power). The spectra of cells were obtained with 0.5 s for each measured point of Raman image. The Raman spectra obtained from LDs of EC treated with AXT were compared to the reference spectra of AXT in the solid state and dissolved in castor oil, using 3 mW laser power, 0.5 s integration time and 10 accumulations that were averaged in order to present one spectrum. 

### 3.3. Data Analysis

Data processing was carried out using WITec Project Plus software. All obtained spectra were processed by a routine cosmic ray removal and were baseline corrected using autopolynomial of degree 3. Cluster analysis (CA) was performed with *k*-means method using the Manhattan distance (WITec Project Plus software) to group classes and extract average spectra representing cell organelles. The average spectra of cells were obtained by averaging the spectra of the whole cells from 5 different cells (*n* = 5) for each group, plotted with standard deviation using Origin Pro 2020b (OriginLab Corporation) software.

## 4. Conclusions

Lipids can be visualized directly in cells by Raman imaging due to their characteristic spectral profile and relatively large scattering cross-section, but a labeled approach using AXT has a significant advantage. The latter measurement can be performed with a laser power 10 times lower, and the signal assigned to lipids is much stronger. In this work, AXT was tested for visualization of lipids in EC of various vascular beds, and it has proved to be a universal molecular reporter for lipid visualization. Moreover, the contrast of Raman images based on the AXT marker band (1530–1500 cm^−1^) is even better than that for images obtained by an integration of the marker bands of lipids (2900–2830 cm^−1^).

It is worth mentioning that the presented method of utilizing AXT as a Raman probe for lipids in EC is limited to the tracking of lipid distribution in cells, as it is challenging to accurately quantify the amount of lipids in the samples based on Raman spectra dominated solely by AXT resonance bands. Moreover, the presented method does not provide information on the composition of LDs in EC due to the tendency of AXT to accumulate in lipids of EC regardless of their class. Compositional information on LDs could be beneficial in studying the development of ED, what is achievable by the label-free Raman approach. However, the presented labeled approach to detect lipids in EC allows for the reduction of undesirable effects associated with higher laser power, especially on relatively fragile samples, representing an advantage over the conventional Raman imaging method. AXT enables better visualizing of lipids in ECs while avoiding the undesirable effects on cell viability and study outcome associated with the cytotoxic and biologically active dyes of widely used methods i.e., fluorescence imaging.

The main conclusion of this work is that AXT is a Raman probe with great potential for detecting cellular lipids in the EC of various organs, hence, it can be used to study endothelial dysfunction, which is reflected in increased lipid deposition.
